# Adjuvant Chemotherapy and Outcomes in Older Adult Patients With Biliary Tract Cancer

**DOI:** 10.1001/jamanetworkopen.2023.51502

**Published:** 2024-01-11

**Authors:** Olumide B. Gbolahan, Xiaojie Zhi, Yuan Liu, Mihir M. Shah, David A. Kooby, Olatunji B. Alese

**Affiliations:** 1 Department of Hematology and Medical Oncology, Emory University School of Medicine, Atlanta, Georgia; 2 Department of Biostatistics and Bioinformatics, Rollins School of Public Health, Emory University, Atlanta, Georgia; 3 Division of Surgical Oncology, Department of Surgery, Emory University School of Medicine, Atlanta, Georgia

## Abstract

**Importance:**

The association of adjuvant chemotherapy (AC) with survival in the general population of patients with resected biliary tract cancer (BTC) remains controversial. As such, the role of this treatment in the treatment of older adult patients (aged ≥70 years) needs to be evaluated.

**Objective:**

To describe the patterns of use of AC and compare survival outcomes of AC and observation in older adult patients following resection of BTC.

**Design, Setting, and Participants:**

This retrospective cohort study included 8091 older adult patients with resected BTC with data available in the National Cancer Database from January 1, 2004, to December 31, 2019. Patients were divided into 2 cohorts: AC and observation. The AC cohort was subdivided into single-agent and multiagent AC treatment.

**Exposures:**

Adjuvant chemotherapy vs observation following BTC resection.

**Main Outcomes and Measures:**

The primary outcome was overall survival (OS) of patients who received AC compared with observation following resection of BTC as evaluated using Kaplan-Meier estimates and multivariable Cox proportional hazards regression models. Inverse probability of treatment weighting and propensity score matching were performed to address indication bias.

**Results:**

Between 2004 and 2019, of 8091 older adult patients with resected BTC identified (median [range] age, 77 [70-90] years; 5136 women [63.5%]; 2955 men [36.5%]), only one-third (2632 [32.5%]) received AC. There was an increase in the use of AC across the study period from 20.7% (n = 495) in 2004 to 2009 to 41.2% (n = 856) in 2016 to 2019. Age 80 years or older (odds ratio, 0.29; 95% CI, 0.25-0.33; *P* < .001) and gallbladder primary site (odds ratio, 0.71; 95% CI, 0.61-0.83; *P* < .001) were associated with a lower odds of AC. Following inverse probability of treatment weighting, as a composite, AC was not associated with improved survival (median OS, 20.5 months; 95% CI, 19.2-21.7 months) compared with observation (median OS, 19.0 months; 95% CI, 18.1-20.3 months). A longer median OS was associated with single-agent AC (21.5 months; 95% CI, 19.9-24.0 months) but not multiagent AC (19.1 months; 95% CI, 17.5-21.1 months) compared with observation (median OS, 17.3 months; 95% CI, 16.1-18.4 months). This improvement in OS with single-agent AC was not apparent on multivariable analysis (hazard ratio [HR], 0.97; 95% CI, 0.89-1.05; *P* = .44). However, age at diagnosis of 80 years or older (HR, 1.35; 95% CI, 1.28-1.42; *P* < .001) and treatment at nonacademic centers (HR, 1.14; 95% CI, 1.07-1.20, *P* < .001) were associated with worse OS.

**Conclusions and Relevance:**

In this cohort study of older adult patients, AC was not associated with an improvement in survival compared with observation following BTC resection. These findings suggest the need for further study of AC for older adult patients who may benefit after curative intent surgery for BTC.

## Key Points

**Question:**

What is the association of adjuvant chemotherapy (AC) after resection of biliary tract cancer with survival in patients aged 70 years or older?

**Findings:**

In this cohort study of 8091 older adult patients who underwent resection of biliary tract cancer, the use of single-agent, but not multiagent, AC improved median overall survival compared with observation alone. However, AC was not associated with overall survival on multivariable analysis.

**Meaning:**

These findings suggest the need for further study of AC for older adult patients who may benefit after curative intent surgery.

## Teaser

This cohort study describes the outcomes of adjuvant chemotherapy vs observation for older adult patients following resection of biliary tract cancer.

## Introduction

Biliary tract cancers (BTCs), including intrahepatic cholangiocarcinoma (ICC), extrahepatic cholangiocarcinoma (ECC), and gallbladder cancer (GBC), although rare, contribute substantially to cancer-related morbidity and mortality in the US.^1^ Biliary tract cancers are largely incurable, as they usually present at an advanced stage. Fewer than 40% of patients present with potentially resectable disease, and among them, approximately 70% experience recurrence.^2,3,4^ Adjuvant chemotherapy (AC) is therefore administered to reduce the risk of recurrence and improve overall survival (OS).

The association of AC with survival in BTC, however, remains controversial. Randomized studies have failed to consistently show a significant OS benefit with AC over observation alone. The Bile Duct Cancer Adjuvant Trial, a randomized study of adjuvant gemcitabine compared with observation in ECC, reported similar survival outcomes in both groups (median OS, 62.3 vs 63.8 months, respectively; hazard ratio [HR], 1.01; 95% CI, 0.70-1.45; *P* = .97).^5^ Furthermore, the addition of oxaliplatin to gemcitabine did not provide survival over observation.^6^ Per-protocol analysis of the Capecitabine Compared With Observation in Resected Biliary Tract Cancer (BILCAP) study showed that adjuvant capecitabine was associated with an improvement in OS compared with observation. However, this finding was not confirmed in the intention-to-treat analysis. Due to the large effect size, capecitabine is now considered a standard of care following resection of BTC.^7^

The median age at diagnosis of BTC in the US is approximately 70 years.^3,8^ Advancing age comes with an increased incidence of comorbid conditions, which overlap with frailty and are associated with poor tolerance of chemotherapy.^9,10,11^ Given the inconsistent benefit of AC in the general population of patients with BTC, it is particularly important to determine the outcomes among older adults (aged ≥70 years) who may experience more adverse events and miss out on the marginal benefit. In this study, we assessed the use and nonuse of AC in a clinical cohort of older patients with BTC to measure differences in survival outcomes.

## Methods

### Cohort Selection

We performed this retrospective cohort study of deidentified data available from the National Cancer Database (NCDB). The NCDB captures approximately 70% of all newly diagnosed cancer cases in the US. We queried the NCDB participant use files from January 1, 2004, to December 31, 2019. This study was conducted with a waiver of informed consent by the Emory University institutional review board because all patient- and hospital-level data in the NCDB are deidentified. This study followed the Strengthening the Reporting of Observational Studies in Epidemiology (STROBE) reporting guideline.

### Study Population

The study included patients aged 70 years or older with BTC based on *International Classification of Diseases for Oncology, Third Edition* morphology codes 8010, 8020, 8030, 8041, 8050, 8070, 8083, 8140, 8160, 8180, 8255, 8260, 8310, 8323, 8480, 8490, 8500, and 8560 and topography codes C239 (gallbladder), C240 (extrahepatic bile duct), and C221 (intrahepatic bile duct). The Facility Oncology Registry Data standard codes 30 to 80 were used to identify patients who underwent resection. Other inclusion criteria were cT2 to cT4 and cN0 to cN2 disease. Patients who did not undergo definitive surgical resection or who had metastatic disease were excluded. We also excluded those who did not receive all first-course treatment at the reporting facility and those with unknown treatment or survival data (eFigure 1 in Supplement 1). Furthermore, we excluded patients who died within 90 days from definitive surgery to account for immortal time bias. Cancers of the ampulla of Vater were excluded because, depending on the histologic subtype, these may be treated like small-bowel cancer.

### Variables

The main variable of interest was AC (single agent and multiagent), and the primary outcome was OS, defined as time from diagnosis until death (for patients who were alive at least 90 days after surgery). We collected data about receipt of AC and divided the overall cohort into 2 groups: observation and AC (which was further subdivided into single agent and multiagent).

We collected clinical and demographic data, including age at diagnosis (dichotomized to <80 and ≥80 years), period of diagnosis, sex, race and ethnicity (Black, White, other [including American Indian, Asian, Hispanic, and Pacific Islander], or unknown), Charlson-Deyo Comorbidity Index, treatment facility type (academic, nonacademic), median income quartiles, and insurance status. Race and ethnicity were identified based on the documentation in the database. In addition, the other category was created to include the very small numbers of individuals who identified as the races and ethnicities listed. Race and ethnicity were included as demographic variables that may be associated with the use of AC. We also collected data on tumor characteristics, including NCDB analytic group stage (pathologic stage group), pathologic tumor stage (pT) and pathologic nodal status (pN), and surgical resection margin (R0, R1, or R2) status.

### Statistical Analysis

Analyses were conducted between May 19 and November 16, 2023. Descriptive statistical analyses were performed for all categorical variables of interest using χ^2^ tests and for numerical variables using *t* test. Univariable and multivariable analysis were conducted to identify factors associated with the use of AC. The parametric *P* value was calculated using analysis of variance for numerical covariates and χ^2^ test for categorical covariates. To assess the associations between patient characteristics and OS, univariable Cox proportional hazards regression was performed, with survival associations expressed as HRs and corresponding 95% CIs. Multivariable Cox proportional hazards regression using backward elimination with an α level of removal of .20 was performed. Kaplan-Meier curves were generated to compare OS between interested patient and treatment characteristic categories using the log-rank test. Both propensity score matching (PSM) and average treatment effect matched weight using inverse probability of treatment weighting (IPTW) methods were conducted to compare OS between patients treated or not treated with AC under relatively homogenous study cohorts in terms of baseline characteristics. Groups were matched based on facility type, surgical resection of the primary site, surgical margins, age at diagnosis, sex, race and ethnicity, annual income, region, comorbidity status, year of diagnosis, and pathologic stage.

All analyses were performed using SAS, version 9.4 software (SAS Institute Inc) and SAS macros developed by the Biostatistics and Bioinformatics and Winship Research Informatics Shared Resources at Winship Cancer Institute in Atlanta, Georgia. A 2-sided *P* < .05 was set as the threshold for significance.

## Results

### Patient Characteristics

We identified 8091 older adult patients (median age, 77 years; range, 70-90 years) diagnosed and treated between 2004 and 2019 (Table 1). The majority of the patients were aged 70 to 80 years (5050 [62.4%]); 5136 (63.5%) were women and 2955 (36.5%) men; and 755 (9.3%) were Black, 6755 (83.5%) White, and 581 (7.2%) other or unknown race and ethnicity. Most patients had a comorbidity index of 0 (5209 [64.4%]), with only 956 (11.8%) having a score of 2 or higher. Only 2632 patients (32.5%) received AC, among whom 1059 (40.2%) received multiagent therapy, 1437 (54.6%) received single-agent therapy, and 136 (5.2%) received an unknown regimen.

**Table 1.** Characteristics of Patients Aged 70 Years or Older Who Underwent Surgery for Biliary Tract Cancer, 2004-2019

Characteristic	Unweighted study population, No. (%)			*P* value
	Overall (N = 8091)	Adjuvant chemotherapy (n = 2632)	Observation (n = 5459)	
Age, median (SD), y	77 (5.7)	75 (4.5)	79 (5.9)	
70-79	5050 (62.4)	2112 (41.8)	2938 (58.2)	<.001
≥80	3041 (37.6)	520 (17.1)	2521 (82.9)	
Sex				
Female	5136 (63.5)	1501 (29.2)	3635 (70.8)	<.001
Male	2955 (36.5)	1131 (38.3)	1824 (61.7)	
Race and ethnicity				
Black	755 (9.3)	250 (33.1)	505 (66.9)	.92
White	6755 (83.5)	2191 (32.4)	4564 (67.6)	
Other or unknown^a^	581 (7.2)	191 (32.9)	390 (67.1)	
Charlson-Deyo Comorbidity Index				
0	5209 (64.4)	1774 (34.1)	3435 (65.9)	<.001
1	1926 (23.8)	583 (30.3)	1343 (69.7)	
≥2	956 (11.8)	275 (28.8)	681 (71.2)	
Primary site				
Intrahepatic bile duct	83 (1.0)	36 (43.4)	47 (56.6)	<.001
Gallbladder	6202 (76.7)	1806 (29.1)	4396 (70.9)	
Extrahepatic bile duct	1806 (22.3)	790 (43.7)	1016 (56.3)	
Pathologic tumor stage				
T1	905 (11.2)	79 (8.7)	826 (91.3)	<.001
T2	3036 (37.5)	902 (29.7)	2134 (70.3)	
T3	2404 (29.7)	1078 (44.8)	1326 (55.2)	
T4	140 (1.7)	68 (48.6)	72 (51.4)	
Not available	1606 (19.8)	505 (31.4)	1101 (68.6)	
Pathologic nodal status				
N0	2836 (35.1)	683 (24.1)	2153 (75.9)	<.001
N1	1791 (22.1)	935 (52.2)	856 (47.8)	
N2	76 (0.9)	51 (67.1)	25 (32.9)	
Not available	3388 (41.9)	963 (28.4)	2425 (71.6)	
Pathologic stage				
0	214 (2.6)	2 (0.9)	212 (99.1)	<.001
I	1084 (13.4)	115 (10.6)	969 (89.4)	
II	2536 (31.3)	808 (31.9)	1728 (68.1)	
III	1340 (16.6)	657 (49.0)	683 (51.0)	
IV	790 (9.8)	415 (52.5)	375 (47.5)	
Not available	2127 (26.3)	635 (29. 9)	1492 (70.2)	
Surgical margins				
R1 or R2	2064 (25.5)	918 (44.5)	1146 (55.5)	<.001
R0	5475 (67.7)	1550 (28.3)	3925 (71.7)	
Not available	552 (6.8)	164 (29.7)	388 (70.3)	
Year of diagnosis				
2004-2009	2390 (29.5)	495 (20.7)	1895 (79.3)	<.001
2010-2015	3624 (44.8)	1281 (35.4)	2343 (64.6)	
2016-2019	2077 (25.7)	856 (41.2)	1221 (58.8)	
Facility location				
Northeast	1933 (23.9)	657 (34.0)	1276 (66.0)	.01
South	2658 (32.9)	855 (32.2)	1803 (67.8)	
Midwest	2097 (25.9)	711 (33.9)	1386 (66.1)	
West	1403 (17.3)	409 (29.2)	994 (70.9)	
Facility type				
Academic	2926 (36.2)	1058 (36.2)	1868 (63.8)	<.001
Nonacademic	5165 (63.8)	1574 (30.5)	3591 (69.5)	
Income level, $				
≥46 000	3056 (37.8)	1267 (30.8)	2842 (69.2)	<.001
<46 000	4109 (50.7)	1019 (33.3)	2037 (66.7)	
Not available	926 (11.4)	346 (37.4)	580 (62.6)	
Primary payer				
Private	843 (10.4)	287 (34.1)	556 (66.0)	.008
Medicaid	203 (2.5)	49 (24.1)	154 (75.9)	
Medicare or other government	6860 (84.8)	2249 (32.8)	4611 (67.2)	
Not insured or unknown	185 (2.3)	47 (25.4)	138 (74.6)	

^a^ Other race and ethnicity included American Indian, Asian, Hispanic, and Pacific Islander.

Only 520 patients (17.1%) aged 80 years or older received AC, while 2112 (41.8%) aged 70 to 79 years did (*P* < .001). There was an increase in the use of AC with increasing pT stage, with 8.7% of patients (n = 79) with T1 tumors receiving AC and up to 48.6% (n = 68) with pT4 tumors receiving AC (*P* < .001). A higher proportion of patients with positive pN status (pN1, 52.2% [n = 935]; pN2, 67.1% [n = 51]) received AC, while only 24.1% (n = 683) of those with pN0 did (*P* < .001). Accordingly, there was an increase in AC receipt with advanced TNM pathologic stage (stage I, 10.6% [n = 115]; stage IV, 52.5% [n = 415]; *P* < .001). A higher proportion of patients with positive surgical margins (R1 or R2) received AC (44.5% [n = 918] vs 28.3% [n = 1550] with R0 resection).

### Trends in the Use of AC

**Figure 1.**
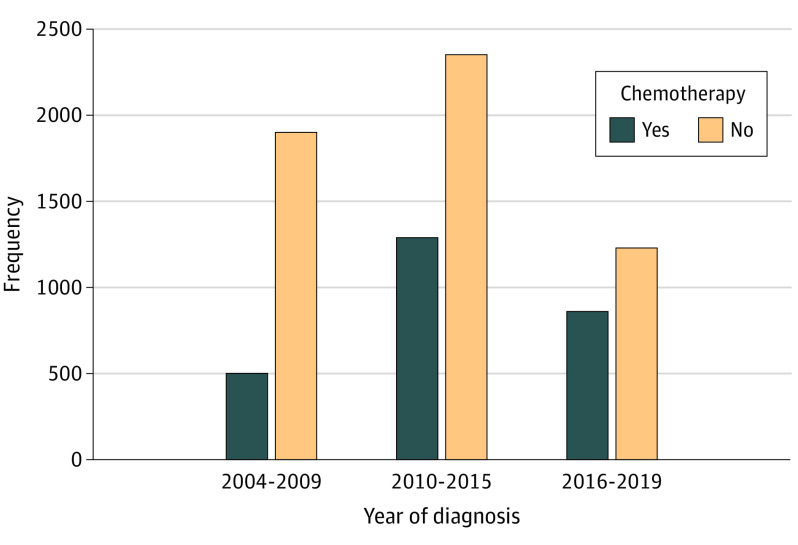
Trends in Adjuvant Chemotherapy Use Among Patients Aged 70 Years or Older Who Underwent Surgery for Biliary Tract Cancer, 2004-2019

There was an increase in the use of AC across the study period, with 20.7% (n = 495) of the cohort receiving AC in 2004 to 2009 compared with 41.2% (n = 856) in the most recent period of 2016 to 2019. Multiagent AC use in this cohort declined after 2015, while single-agent AC use increased (Figure 1; eFigure 2 in Supplement 1).

Race and ethnicity and treatment in a rural vs urban setting were not associated with the use of AC and were excluded from multivariable logistic regression modeling. Diagnosis at age 80 years or older (odds ratio [OR], 0.29; 95% CI, 0.25-0.33; *P* < .001), gallbladder primary site (OR, 0.71; 95% CI, 0.61-0.83; *P* < .001), and Medicaid as primary payer (OR, 0.57; 95% CI, 0.38-0.85; *P* = .006) were associated with lower odds of receiving AC. The year of diagnosis, advanced TNM stage, positive surgical margins, and use of radiation therapy increased the odds of receiving AC (Table 2).

**Table 2.** Multivariable Logistic Regression Modeling of Factors That Determine Receipt of Adjuvant Chemotherapy

Covariate	No. of patients	OR (95% CI)^a^	OR *P* value	Type 3 *P* value^b^
Radiation				
Yes	1304	20.17 (16.85-24.15)	<.001	<.001
No	6787	1 [Reference]	NA	
Facility location				
Northeast	1933	1.19 (1.02-1.40)	.03	.001
Midwest	2097	1.14 (0.97-1.33)	.11	
West	1403	0.84 (0.71-1.01)	.06	
South	2658	1 [Reference]	NA	
Primary site				
Intrahepatic bile duct	83	0.83 (0.48-1.45)	.52	<.001
Gallbladder	6202	0.71 (0.61-0.83)	<.001	
Extrahepatic bile duct	1806	1 [Reference]	NA	
Age at diagnosis, y				
≥80	3041	0.29 (0.25-0.33)	<.001	<.001
<80	5050	1 [Reference]	NA	
Primary payer				
Not insured or unknown	185	0.71 (0.47-1.08)	.11	.009
Private	843	1.10 (0.92-1.33)	.30	
Medicaid	203	0.57 (0.38-0.85)	.006	
Medicare or other government	6860	1 [Reference]	NA	
Charlson-Deyo Comorbidity Index				
0	5209	1 [Reference]	NA	<.001
1	1926	0.82 (0.71-0.94)	.005	
≥2	956	0.75 (0.62-0.90)	.002	
Year of diagnosis				
2004-2009	2390	1 [Reference]	NA	<.001
2010-2015	3624	2.28 (1.92-2.71)	<.001	
2016-2019	2077	3.77 (3.12-4.54)	<.001	
Pathologic tumor stage				
T1	905	1 [Reference]	NA	<.001
T2	3036	1.75 (1.28-2.39)	<.001	
T3	2404	2.27 (1.63-3.15)	<.001	
T4	140	2.08 (1.24-3.51)	.006	
Not available	1606	2.23 (1.58-3.15)	<.001	
Pathologic nodal status				
N0	2836	1 [Reference]	NA	
N1	1791	1.91 (1.61-2.27)	<.001	<.001
N2	76	2.15 (1.20-3.83)	.01	
Not available	3388	1.28 (1.06-1.55)	.009	
Pathologic stage				
0	214	0.13 (0.03-0.56)	.006	<.001
I	1084	1 [Reference]	NA	
II	2536	1.76 (1.32-2.36)	<.001	
III	1340	2.50 (1.78-3.52)	<.001	
IV	790	5.08 (3.61-7.16)	<.001	
Not available	2127	1.59 (1.14-2.20)	.006	
Surgical margins				
Yes	2064	1.47 (1.28-1.69)	<.001	<.001
No	5475	1 [Reference]	NA	
Not available	552	1.20 (0.94-1.52)	.14	

Abbreviations: NA, not applicable; OR, odds ratio.

^a^ Number of observations in the original data set was 8091. Number of observations used was 8091.

^b^ Backward selection with an α level of removal of .05 was used.

### AC vs Observation-Alone Outcomes

**Figure 2.**
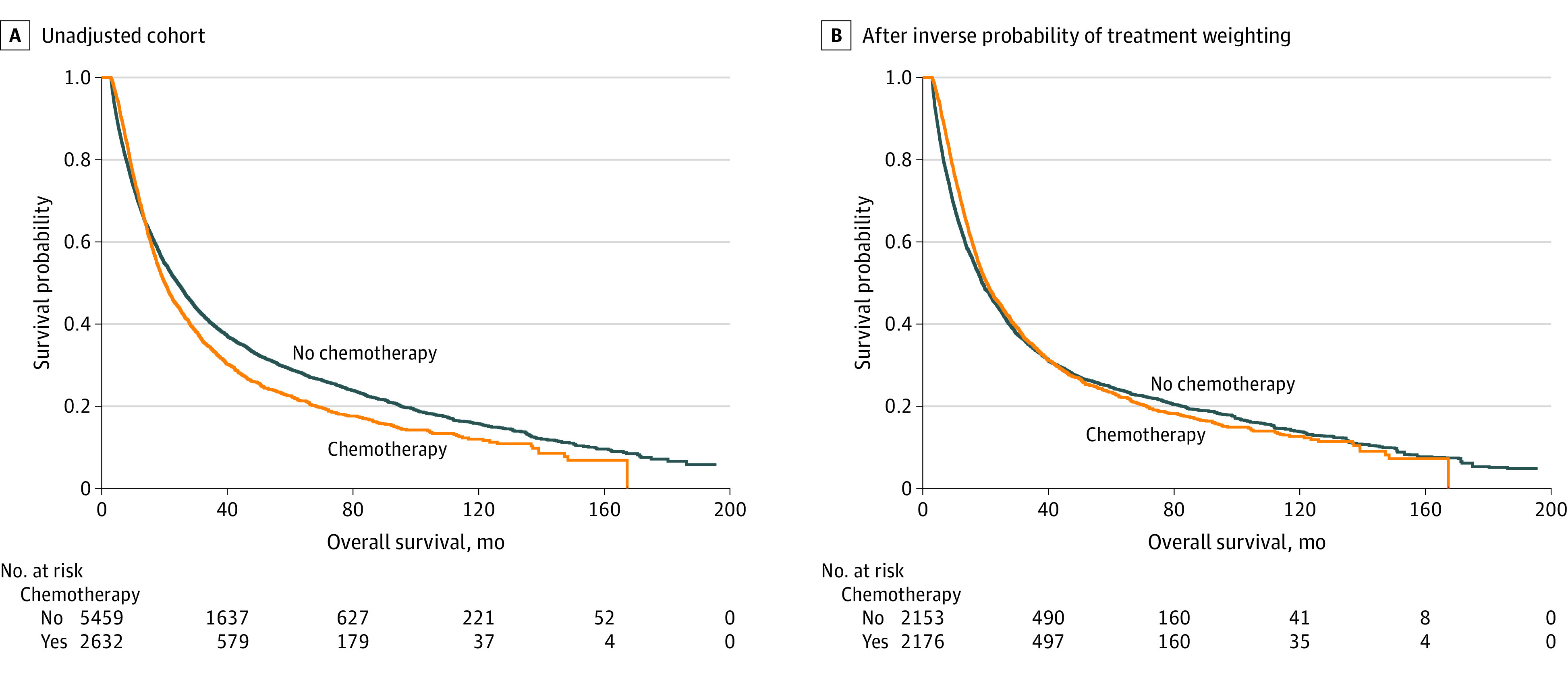
Kaplan-Meier Survival Analysis by Treatment Group

After a median follow-up of 21 months (range, 3-196 months), AC was not associated with an improvement in survival compared with observation alone (HR, 0.98; 95% CI, 0.91-1.05; *P* = .51). The median OS was 24.3 months (95% CI, 23.0-25.5 months) in the observation cohort vs 20.2 months (95% CI, 19.1-21.3; *P* < .001) in the AC cohort. While there was no difference in the 12-month survival rates between the 2 groups (observation: 69.1% [95% CI, 67.8%-70.3%]; AC: 70.4% [95% CI, 68.6%-72.1%]), the 5-year survival rates were 29.1% (95% CI, 27.8%-30.4%) and 22.6% (95% CI, 20.8%-24.4%), respectively (Figure 2A).

Following IPTW adjustment, the standardized difference for variables was less than 10%, indicating that patients in both the AC and observation-only cohorts were comparable (eTable and eFigure 3 in Supplement 1). The propensity score distribution between the 2 groups also achieved adequate balance (eFigure 4 in Supplement 1). Following weighting, the Cox proportional hazards regression analysis showed no difference in median OS between the AC and observation cohorts (HR, 0.96; 95% CI, 0.9-1.02; *P* = .14). The median survival was 19.0 months (95% CI, 18.1-20.3 months) in the observation cohort and 20.5 months (95% CI, 19.2-21.7 months) in the AC cohort (*P* = .12). There was also no difference in the 5-year survival rate in the observation cohort (24.6%; 95% CI, 23.1%-26.2%) compared with the AC cohort (23.4%; 95% CI, 21.6%-25.4%) (Figure 2B). The PSM analysis corroborated this result (eFigure 5 in Supplement 1).

We queried whether there would be a difference in survival based on the receipt of single-agent vs multiagent AC. In the unweighted analysis, single-agent AC was associated with a median OS of 21.5 months (95% CI, 20.0-24.2 months) vs 18.3 months (95% CI, 17.2-19.8 months) with multiagent AC and 24.3 months (95% CI, 23.0-23.5 months) with observation. The 5-year survival rate was 25.7% (95% CI, 23.2%-28.2%) vs 18.2% (95% CI, 15.6%-20.9%) and 29.1% (95% CI, 27.8%-30.4%) for single-agent AC, multiagent AC, and observation, respectively. The benefit of single-agent AC persisted after weighting. The median OS was 21.5 months (95% CI, 19.9-24.0 months) with single-agent AC vs 19.1 months (95% CI, 17.5-21.1 months) with multiagent AC and 17.3 months (95% CI, 16.1-18.4 months) with observation (eFigure 6 in Supplement 1). Compared with observation, single-agent AC (HR, 0.85; 95% CI, 0.78-0.93; *P* < .001) but not multiagent AC (HR, 0.96; 95% CI, 0.88-1.05; *P* = .34) was associated with an improvement in OS on Kaplan-Meier analysis.

### Factors Associated With OS

Over the 15-year study period, there was an improvement in median OS of approximately 9 months (2004-2009: median OS, 18.6 months [95% CI, 17.4-19.7 months]; 2010-2015: median OS, 23.4 months [95% CI, 21.9-24.9 months]; 2016-2019: median OS, 27.9 months [95% CI, 25.9-29.7 months]; *P* < .001) (eFigure 7 in Supplement 1). Compared with observation only, the receipt of AC (HR, 1.14; 95% CI, 1.08-1.20 *P* < .001), age at diagnosis of 80 years or older (HR, 1.31; 95% CI, 1.24-1.38; *P* < .001), and treatment at nonacademic centers (HR, 1.17; 95% CI, 1.11-1.23; *P* < .001) were associated with inferior survival outcomes. On multivariable analysis, observation vs AC was no longer associated with survival (HR, 0.98; 95% CI, 0.91-1.05; *P* = .51). Additionally, single-agent chemotherapy was no longer associated with OS (HR, 0.97; 95% CI, 0.89-1.05; *P* = .44). Age at diagnosis of 80 years or older (HR, 1.35; 95% CI, 1.28-1.43; *P* < .001) and treatment at nonacademic centers (HR, 1.13; 95% CI, 1.06-1.19; *P* < .001) remained associated with worse OS. The periods of diagnosis 2010 to 2015 (HR, 0.77; 95% CI, 0.72-0.82) and 2016 to 2019 (0.68; 95% CI, 0.63-0.74) continued to be associated with improvement in OS compared with 2004 to 2009 (*P* < .001) (Table 3).

**Table 3.** Multivariable Logistic Regression of Factors Associated With Overall Survival

Covariate	Overall survival, mo		
	HR (95% CI)^a^	HR *P* value	Type 3 *P* value^b^
Chemotherapy			
Yes	0.98 (0.91-1.05)	.51	.51
No	1 [Reference]	NA	
No. of chemotherapy agents			
Single agent	0.97 (0.89-1.05)	.44	.74
Multiple agents	0.98 (0.90-1.07)	.70	
None	1 [Reference]	NA	
Radiation			
Yes	0.85 (0.79-0.93)	<.001	<.001
No	1 [Reference]	NA	
Facility type			
Nonacademic	1.13 (1.06-1.19)	<.001	<.001
Academic	1 [Reference]	NA	
Facility location			
Northeast	0.88 (0.81-0.94)	<.001	<.001
Midwest	1.04 (0.97-1.12)	.24	
West	0.96 (0.89-1.04)	.31	
South	1 [Reference]	NA	
Primary site			
Intrahepatic bile duct	0.75 (0.56-1.00)	.051	.11
Gallbladder	1.02 (0.95-1.09)	.66	
Extrahepatic bile duct	1 [Reference]	NA	
Age at diagnosis, y			
≥80	1.35 (1.28-1.43)	<.001	<.001
<80	1 [Reference]	NA	
Race			
Black	1.05 (0.96-1.15)	.31	.14
White	1 [Reference]	NA	
Other or unknown^c^	0.92 (0.82-1.02)	.11	
Primary payer			
Not insured or unknown	0.95 (0.79-1.14)	.55	.006
Private	0.88 (0.81-0.96)	.004	
Medicaid	0.82 (0.67-0.99)	.04	
Medicare or other government	1 [Reference]	NA	
Median income, $			
<46 000	1.07 (0.99-1.15)	.07	.07
≥46 000	1 [Reference]	NA	
Not available	0.94 (0.86-1.04)	.22	
Charleson-Deyo Comorbidity Index			
0	1 [Reference]	NA	<.001
1	1.11 (1.05-1.18)	<.001	
≥2	1.28 (1.18-1.39)	<.001	
Year of diagnosis			
2004-2009	1 [Reference]	NA	<.001
2010-2015	0.77 (0.72-0.82)	<.001	
2016-2019	0.68 (0.63-0.74)	<.001	
Pathologic tumor stage			
T1	1 [Reference]	NA	<.001
T2	1.31 (1.17-1.46)	<.001	
T3	1.85 (1.64-2.09)	<.001	
T4	1.31 (1.05-1.64)	.02	
Not available	1.47 (1.30-1.67)	<.001	
Pathologic nodal status			
N0	1 [Reference]	NA	<.001
N1	1.23 (1.14-1.33)	<.001	
N2	0.93 (0.70-1.22)	.58	
Not available	1.36 (1.26-1.47)	<.001	
Pathologic stage			
0	0.67 (0.55-0.82)	<.001	<.001
I	1 [Reference]	NA	
II	1.18 (1.06-1.30)	.002	
III	1.43 (1.25-1.63)	<.001	
IV	2.31 (2.02-2.63)	<.001	
Not available	1.17 (1.05-1.31)	.005	
Surgical margins			
Yes	1.76 (1.75-1.98)	<.001	<.001
No	1 [Reference]	NA	
Not available	1.51 (1.36-1.67)	<.001	

Abbreviations: HR, hazard ratio; NA, not applicable.

^a^ Number of observations in the original data set was 8091. Number of observations used was 8091.

^b^ Backward selection with an α level of removal of .20 was used. No variables were removed from the model.

^c^ Other race and ethnicity included American Indian, Asian, Hispanic, and Pacific Islander.

## Discussion

Due to concerns about age-related comorbidities, competing mortality risks, and the potential for increased toxic effects with AC in older adults,^12,13^ we hypothesized in this cohort study that AC would not provide a survival benefit in older adult patients with resected BTC. In our analysis, AC was administered to only one-third of older adult patients between 2004 and 2019, although its use increased over time. Single-agent chemotherapy provided an improvement in survival over observation alone, but overall, AC was not associated with an improvement in survival on multivariable analysis. These results suggest that a subgroup of older adult patients with resected BTC may benefit from AC, the role of which still needs to be explored further among this cohort.

The BILCAP study established capecitabine as the standard-of-care AC for BTC in Western Europe and the US. However, BILCAP is limited in its generalizability to an older adult population given that the median age in that study was 62 years (IQR, 55-69 years).^10^ Nevertheless, more recent data from the Japanese phase III Adjuvant S-1 vs Observation for Resected Biliary Tract Cancer (ASCOT) trial strengthen the case for the use of adjuvant fluorouracil for BTC.^14^ In ASCOT, 440 patients aged 20 to 80 years were randomized to adjuvant S-1, a mixture of tegafur (a fluorouracil prodrug), gimeracil (inhibitor of dihydropyrimidine dehydrogenase), and oteracil potassium or observation. Adjuvant S-1 was associated with an improvement in OS (HR, 0.69; 95% CI, 0.51-0.94) compared with observation. The median age of patients in the ASCOT trial was 68 years (IQR, 33-80 years), closer to the age distribution of similar clinical patients in the US.

The phase III PRODIGE 34 ADAGE study of older adults (aged ≥70 years) who received adjuvant fluoropyrimidine for stage III colon cancer highlights the challenges of administering AC in this population.^15^ The rate of grade 3 to 5 toxic effects was approximately 26% in fit (as assessed by frailty score rather than comorbidity score) patients who received adjuvant 5-fluorouracil or capecitabine and up to 40% in less-fit patients. While 16% of fit patients who received fluoropyrimidine stopped treatment, 38% of less-fit patients stopped fluoropyrimidine early. Capecitabine was administered to 50% of the less-fit patients. The investigators speculated that capecitabine may be more difficult to tolerate by older adults.

Contrary to our hypothesis and despite a median age at diagnosis of 77 years, 64.4% of patients had a Charlson-Deyo Comorbidity Index score of 0, suggesting that no traditional comorbidities were reported. It is reasonable that older patients who underwent surgical resection would have had potentially fewer comorbid conditions; however, only approximately 33% of these patients received AC between 2015 and 2019. With this data set, we are thus unable to draw any meaningful inference about how the tolerability of AC may be associated with survival among older adults.

The low use of AC, on the other hand, is consistent with published studies. Ozer et al^16^ reported that AC was administered to approximately one-half (49%) of all patients with gallbladder cancer in an analysis of NCDB data reflecting the practice between 2004 and 2016. In their study, AC was associated with an improvement in OS compared with surgery alone (22 vs 18 months; HR, 0.78; 95% CI, 0.63-0.96). They highlighted that surgery alone was more widely used in older adult patients (median age, 72 years; IQR, 63-81 years) compared with AC or neoadjuvant chemotherapy but did not report the proportion of older adults who received AC vs observation. Another NCDB analysis found that AC was used in only 29% of patients following resection of GBC.^17^ In accordance with our findings, OS was not associated with AC (HR, 1.01; 95% CI, 0.92-1.10). The median age at diagnosis was 72 years (IQR, 62-80 years), and 47% (n = 809) of patients in this analysis were older adults (aged ≥70 years). These 2 studies may differ based on inclusion criteria and case definition. The Ozer et al^16^ study included more advanced (T4) tumors, which may benefit more from AC. Furthermore, the latter study included patients diagnosed within an earlier period (2004-2011). Nevertheless, in an investigation of the Surveillance, Epidemiology, and End Results and Medicare–linked database of patients aged 65 years or older with resected GBC, similar to our report, only 25% of patients received AC, and this did not provide a survival benefit over observation (median OS, 17.6 vs 19.5 months; *P* = .77).^18^ Another analysis of NCDB data concluded that adjuvant therapy was associated with improved survival for hilar cholangiocarcinoma (40.0 vs 30.6 months; *P* = .03) but not distal cholangiocarcinoma (33.0 vs 30.3 months; *P* = .12).^19^ Another interesting but not surprising finding in this study was that single-agent AC provided an improvement in median OS compared with observation and multiagent AC, which may reflect the ineffectiveness of the combination therapy regimens that have been used in the adjuvant setting to date. Alternatively, the finding may reflect a situation where combination therapy truly does not improve survival (over single-agent therapy) in older adult patients, as has been described for colon cancer. In a subgroup analysis of the MOSAIC (Multicenter International Study of Oxaliplatin, Fluorouracil, and Leucovorin in the Adjuvant Treatment of Colon Cancer) study, the addition of oxaliplatin to fluoropyrimidine in the adjuvant setting for colon cancer did not provide a survival benefit for patients aged 70 to 75 years over single-agent 5-fluorouracil.^20^

### Limitations

Our study is limited by its retrospective nature. We attempted to adjust for confounders, an inherent challenge in these studies, by performing an IPTW analysis and PSM, which we recognize do not completely address the problem. However, it was encouraging that the survival analysis from both the IPTW and PSM analyses were in concordance. We also note the negligible number of patients with ICC in this study based on the stringent inclusion and exclusion criteria we used that only captured patients who received surgical resection of potentially curative intent. Nevertheless, given that the current and near-future approaches to AC in ICC may remain similar to ECC and GBC, our findings may be extrapolated with some confidence to ICC. In addition, the NCDB does not capture data about specific chemotherapy regimens. We are therefore unable to determine the multiagent AC treatments administered or, more importantly, whether the single-agent AC used was 5-fluorouracil, capecitabine, or gemcitabine, making it impossible to offer any recommendations about specific agents. Finally, it is important to note that the NCDB attributes a comorbidity index score of 0 to patients whose comorbid conditions are not recorded in the originating medical records. It is therefore possible that the comorbidity of patients is misrepresented,^21^ which may explain why only one-third of patients received AC. It is reasonable to argue that we do not see a benefit for AC in older adults because of this low uptake. We are unable to comment on this possibility on the basis of the available data. A broad view of the AC landscape for BTC would indicate that AC provides marginal survival benefit. Our data suggest that AC may not provide an OS benefit over surgery alone in older adults. If older adults are offered AC, then single-agent chemotherapy should be the preferred option and would be in line with the standard of care, especially as suggested by the phase II STAMP study that the AC combination of gemcitabine and cisplatin may not improve survival compared with single-agent capecitabine for resected ECC.^22^ The ACTICCA-1 (Adjuvant Chemotherapy With Gemcitabine and Cisplatin Compared to Standard of Care After Curative Intent Resection of Biliary Tract Cancer) study (NCT02170090), a large phase III trial, is also evaluating the role of gemcitabine and cisplatin compared with capecitabine in the adjuvant setting for BTC. The results of this trial could provide a more definite answer. Although analysis based on age is not a prespecified end point in the ACTICCA-1 study, an evaluation of differences in outcomes based on age would be important.

## Conclusions

In this cohort study of older adult patients with resected BTC, we present comprehensive data about the association of AC with OS. Although AC is underused in this population, we found that it was not associated with an OS benefit compared with observation alone. These findings suggest the need for further study of AC for older adult patients who may benefit after curative intent surgery.

## Notes

Supplement 1. **eFigure 1.** Criteria for Selection of Patients Who Underwent Surgical Resection for Biliary Tract Cancer **eFigure 2.** Trends in the Use of Single-Agent or Multiagent Adjuvant Chemotherapy, 2004-2019 **eFigure 3.** Population Distribution Before and After Inverse Probability of Treatment Weighting (IP)TW **eFigure 4.** Population Distribution After Propensity Score Matching **eFigure 5.** Kaplan-Meier Survival After Propensity Score Matching **eFigure 6.** Kaplan-Meier Survival Analysis by Treatment Group **eFigure 7.** Kaplan-Meier Survival Across Different 5-Year Time Periods **eTable.** Overall Sample Distribution and Balance Check Before and After Propensity Score Average Treatment Effect Matched Weight Using Inverse Probability of Treatment Weighting

Supplement 2.
Data Sharing Statement
