# Supplementary material for: Adjuvant Chemotherapy and Outcomes in Older Adult Patients With Biliary Tract Cancer
Source: JAMA Netw Open. 2024 Jan 11;7(1):e2351502. doi: 10.1001/jamanetworkopen.2023.51502 (PMC10784855; doi:10.1001/jamanetworkopen.2023.51502)
Supplement: Supplement 1. — eFigure 1. Criteria for Selection of Patients Who Underwent Surgical Resection for Biliary Tract Cancer eFigure 2. Trends in the Use of Single-Agent or Multiagent Adjuvant Chemotherapy, 2004-2019 eFigure 3. Population Distribution Before and After Inverse Probability of Treatment Weighting (IP)TW eFigure 4. Population Distribution After Propensity Score Matching eFigure 5. Kaplan-Meier Survival After Propensity Score Matching eFigure 6. Kaplan-Meier Survival Analysis by Treatment Group eFigure 7. Kaplan-Meier Survival Across Different 5-Year Time Periods eTable. Overall Sample Distribution and Balance Check Before and After Propensity Score Average Treatment Effect Matched Weight Using Inverse Probability of Treatment Weighting [file jamanetwopen-e2351502-s001.pdf]

Supplemental Online Content

Gbolahan OB, Zhi X, Liu Y, Sha MM, Kooby DA, Alesse OB. Adjuvant chemotherapy and outcomes in older adult patients with biliary tract cancer. *JAMA Netw Open*. 2024;7(1):e2351502. doi:10.1001/jamanetworkopen.2023.51502

eFigure 1. Criteria for Selection of Patients Who Underwent Surgical Resection for Biliary Tract Cancer

eFigure 2. Trends in the use of Single-Agent or Multiagent Adjuvant Chemotherapy, 2004-2019

eFigure 3. Population Distribution Before and After Inverse Probability of Treatment Weighting (IP)TW

eFigure 4. Population Distribution After Propensity Score Matching

eFigure 5. Kaplan-Meier Survival After Propensity Score Matching

eFigure 6. Kaplan-Meier Survival Analysis by Treatment Group

eFigure 7. Kaplan-Meier Survival Across Different 5-Year Time Periods

eTable. Overall Sample Distribution and Balance Check Before and After Propensity Score Average Treatment Effect Matched Weight Using Inverse Probability of Treatment Weighting

This supplemental material has been provided by the authors to give readers additional information about their work.

© 2024 Gbolahan OB et al. *JAMA Network Open.*

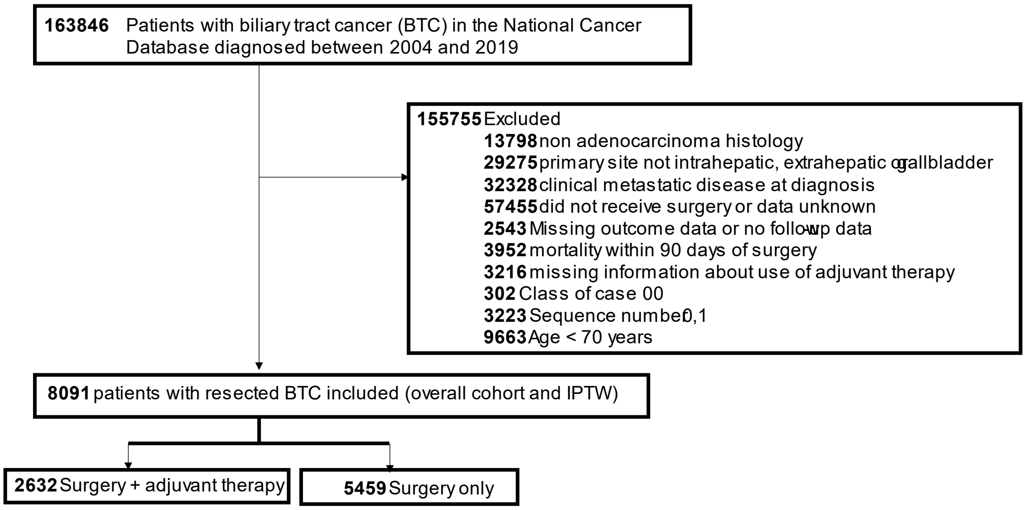

Class of case 00: diagnosis at the reporting facility but treatment or decision not to treat done elsewhere.

eFigure 1: Criteria for selection of patients who underwent surgical resection for biliary tract cancer

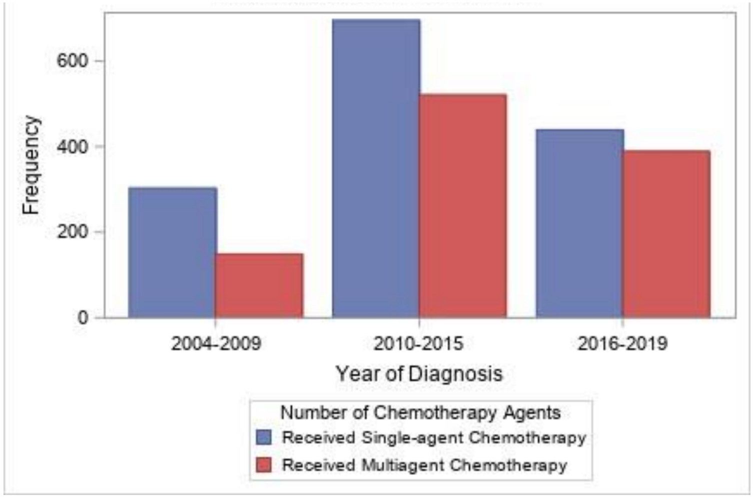

eFigure 2: Trends in the use of single agent or multiagent adjuvant chemotherapy 2004 - 2019

© 2024 Gbolahan OB et al. *JAMA Network Open.*

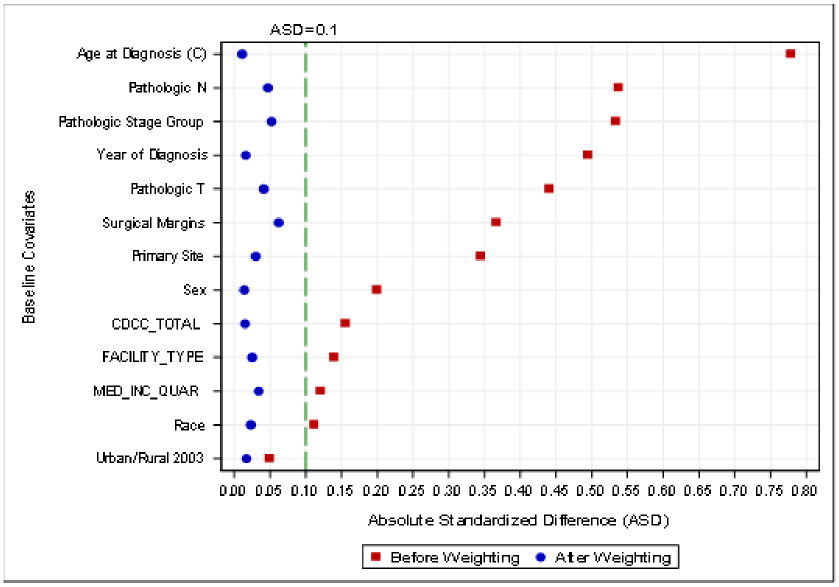

CDCC- Charlson-Deyo Comorbidity Index, Med_Inc_Quar - median income quartiles

eFigure3: Population distribution before and after inverse probability of treatment weighting (IPTW)

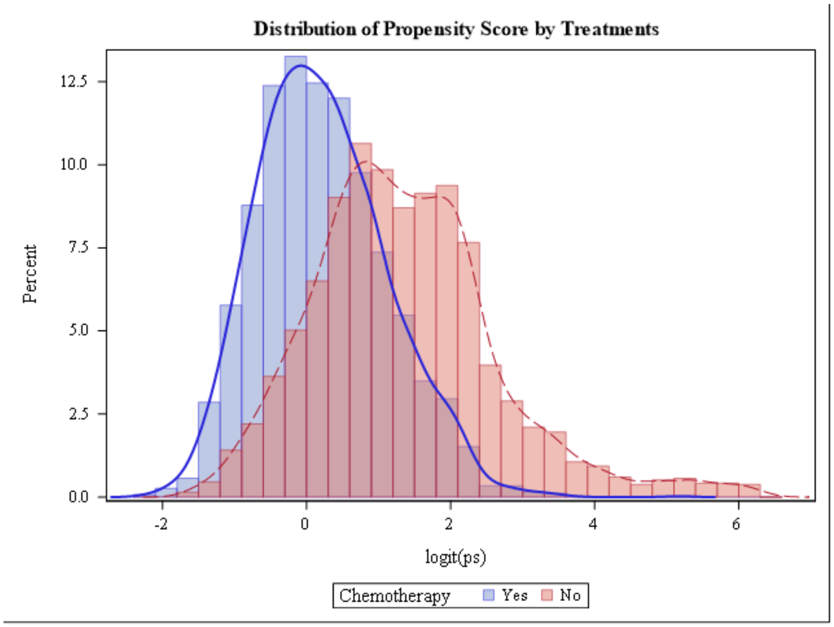

eFigure 4: Population distribution after propensity score matching

© 2024 Gbolahan OB et al. *JAMA Network Open.*

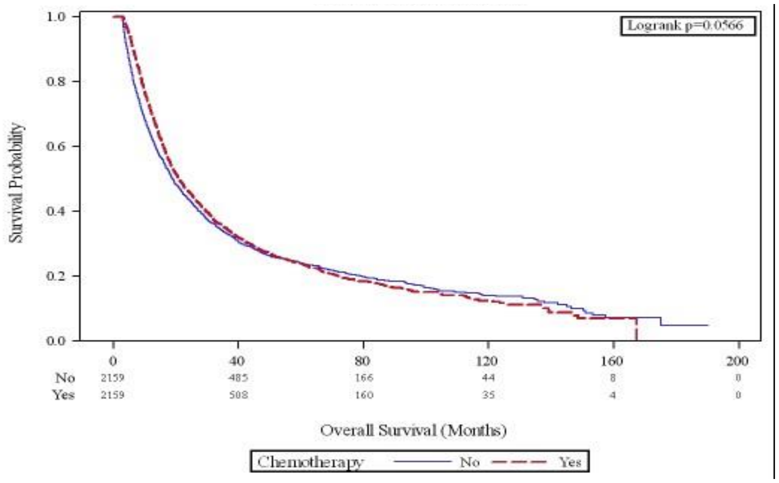

eFigure 5: Kaplan-Meier Survival after propensity score matching

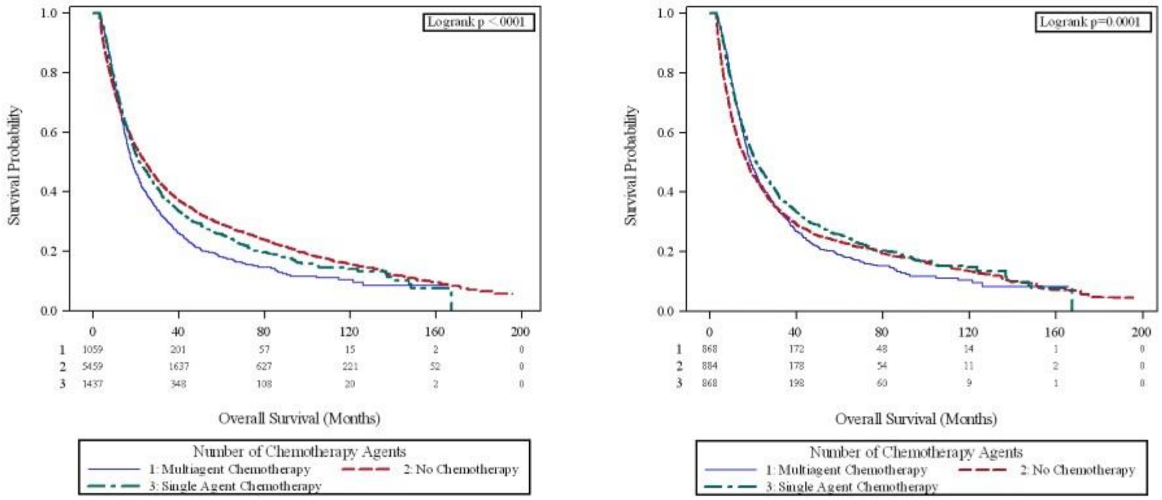

eFigure 6: Kaplan-Meir survival analysis by treatment group. A for the unadjusted cohort. B. After Inverse Probability of Treatment Weighting

© 2024 Gbolahan OB et al. *JAMA Network Open.*

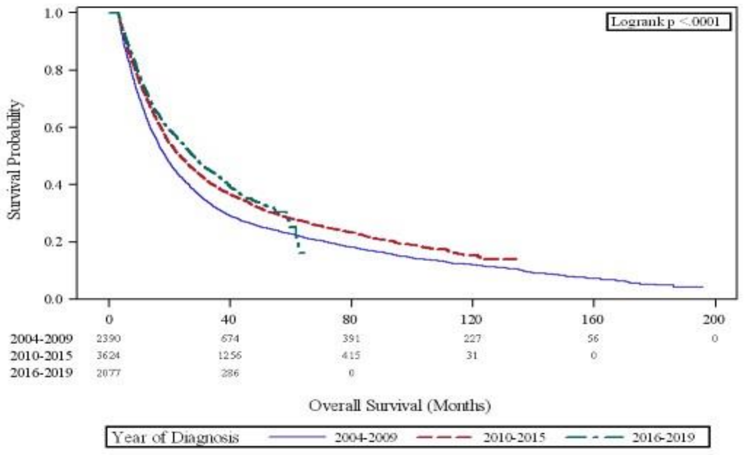

eFigure 7: Kaplan-Meier Survival across different 5 -year time periods

Covariate	Level	Study Sample Distribution		Absolute Standardized Difference(ASD)	
		Before	After	Before	After
Number of Chemotherapy Agents	Overall	7955 (100.0)	2647 (100.0)	-	-
	No_Chemotherapy_Received	5459 (68.6)	891 (33.7)	-	-
	Received_Single_agent_Chemothera	1437 (18.1)	878 (33.2)	-	-
	Received_Multiagent_Chemotherapy	1059 (13.3)	878 (33.2)	-	-
FACILITY_TYPE	Academic/Research Program	2883 (36.2)	1087 (41.1)	**0.14**	0.025
	Non-Academic/Research Program	5072 (63.8)	1560 (58.9)	**0.14**	0.025
Primary Site	C221-Intrahepatic bile duct	81 (1)	33 (1.2)	0.056	0.009
	C239-Gallbladder	6105 (76.7)	1856 (70.1)	**0.345**	0.028
	C240-Extrahepatic bile duct	1769 (22.2)	759 (28.7)	**0.336**	0.03
Age at Diagnosis (C)	>=80	3009 (37.8)	410 (15.5)	**0.779**	0.011
	<80	4946 (62.2)	2237 (84.5)	**0.779**	0.011

© 2024 Gbolahan OB et al. *JAMA Network Open.*

Covariate	Level	Study Sample Distribution		Absolute Standardized Difference(ASD)	
		**Before**	**After**	**Before**	**After**
Sex	Male	2889 (36.3)	1133 (42.8)	**0.2**	0.014
	Female	5066 (63.7)	1515 (57.2)	**0.2**	0.014
Race	White	6641 (83.5)	2219 (83.8)	**0.101**	0.017
	Black	740 (9.3)	226 (8.5)	**0.112**	0.023
	Others/Unknown	574 (7.2)	202 (7.6)	0.02	0.009
MED_INC_QUAR	< $46,000	4031 (50.7)	1274 (48.1)	**0.101**	0.034
	$46,000 +	3010 (37.8)	1005 (38)	0.074	0.022
	Not Available	914 (11.5)	368 (13.9)	**0.121**	0.018
Urban/Rural 2003	Metro	6474 (81.4)	2136 (80.7)	0.038	0.005
	Urban	1060 (13.3)	376 (14.2)	0.03	0.007
	Rural	144 (1.8)	44 (1.6)	0.029	0.004
	Not Available	277 (3.5)	92 (3.5)	0.05	0.017
CDCC_TOTAL	0	5123 (64.4)	1812 (68.4)	**0.156**	0.006
	1	1894 (23.8)	570 (21.5)	0.094	0.011
	2+	938 (11.8)	265 (10)	**0.11**	0.015
Year of Diagnosis	2004-2009	2347 (29.5)	416 (15.7)	**0.495**	0.016
	2010-2015	3559 (44.7)	1312 (49.6)	**0.126**	0.008
	2016-2019	2049 (25.8)	919 (34.7)	**0.319**	0.016
	2004-2009	2347 (29.5)	416 (15.7)	**0.495**	0.016
	2010-2015	3559 (44.7)	1312 (49.6)	**0.126**	0.008
	2016-2019	2049 (25.8)	919 (34.7)	**0.319**	0.016
Pathologic T	T1	897 (11.3)	68 (2.6)	**0.441**	0.04
	T2	2991 (37.6)	861 (32.5)	**0.152**	0.018
	T3	2347 (29.5)	1098 (41.5)	**0.401**	0.041
	T4	138 (1.7)	68 (2.6)	**0.127**	0.018
	Not Available	1582 (19.9)	553 (20.9)	0.023	0.026

© 2024 Gbolahan OB et al. *JAMA Network Open.*

Covariate	Level	Study Sample Distribution		Absolute Standardized Difference(ASD)	
		Before	After	Before	After
Pathologic N	N0	2804 (35.2)	610 (23)	**0.418**	0.022
	N1	1746 (21.9)	995 (37.6)	**0.538**	0.047
	N2	76 (1)	49 (1.9)	**0.172**	0.011
	Not Available	3329 (41.8)	994 (37.5)	**0.195**	0.026
Pathologic Stage Group	Stage 0	213 (2.7)	0 (0)	**0.284**	-
	Stage 1	1075 (13.5)	71 (2.7)	**0.534**	0.007
	Stage 2	2491 (31.3)	710 (26.8)	**0.29**	0.026
	Stage 3	1308 (16.4)	747 (28.2)	**0.368**	0.011
	Stage 4	774 (9.7)	416 (15.7)	**0.477**	0.052
	Not Available	2094 (26.3)	704 (26.6)	0.076	0.022
Surgical Margins	Yes	2026 (25.5)	921 (34.8)	**0.367**	0.062
	No	5393 (67.8)	1562 (59)	**0.351**	0.058
	Not Available	536 (6.7)	164 (6.2)	**0.104**	0.008

* The absolute standardized Difference (ASD) >= 0.1 is bold and indicates insufficient balance.

eTable. overall sample distribution and balance check before and after propensity score average treatment effect matched weight using inverse probability of treatment weighting.

© 2024 Gbolahan OB et al. *JAMA Network Open.*
