# Supplementary material for: Adjuvant Chemotherapy and Outcomes in Older Adult Patients With Biliary Tract Cancer
Source: JAMA Netw Open. 2024 Jan 11;7(1):e2351502. doi: 10.1001/jamanetworkopen.2023.51502 (PMC10784855; doi:10.1001/jamanetworkopen.2023.51502)
Supplement: Supplement 2. — Data Sharing Statement [file jamanetwopen-e2351502-s002.pdf]

Data Sharing Statement

Gbolahan. Adjuvant Chemotherapy and Outcomes in Older Adult Patients With Biliary Tract Cancer. *JAMA Netw Open*. Published January 11, 2024.
doi:10.1001/jamanetworkopen.2023.51502

Data
Data available: Yes
Data types: Deidentified participant data, Data dictionary
How to access data: ogbolah@emory.edu
When available: With publication

Supporting Documents
Document types: Statistical/analytic code
How to access documents: ogbolah@emory.edu
When available: With publication

Additional Information
Who can access the data: anyone requesting the data
Types of analyses: for a specified purpose
Mechanisms of data availability: with investigator support
